# The gene expression profile and cell of origin of canine peripheral T-cell lymphoma

**DOI:** 10.1186/s12885-023-11762-w

**Published:** 2024-01-02

**Authors:** Eileen Owens, Lauren Harris, Adam Harris, Janna Yoshimoto, Robert Burnett, Anne Avery

**Affiliations:** Department of Microbiology, Immunology & Pathology; College of Veterinary Medicine and Biomedical Sciences, Colorado State University (EO, LH, AH, JY, RB, AA), 300 W Lake St, Fort Collins, CO 80521 USA

**Keywords:** Lymphoma, T-cell, Cancer, Gene expression, Transcriptomics, RNA-seq, Canine, GATA3, TBX21, Cell of origin, Thymocyte

## Abstract

**Background:**

Peripheral T-cell lymphoma (PTCL) refers to a heterogenous group of T-cell neoplasms with poor treatment responses and survival times. Canine PTCL clinically and immunophenotypically resembles the most common human subtype, PTCL-not otherwise specified (PTCL-NOS), leading to interest in this canine disease as a naturally occurring model for human PTCL. Gene expression profiling in human PTCL-NOS has helped characterize this ambiguous diagnosis into distinct subtypes, but similar gene expression profiling in canine PTCL is lacking.

**Methods:**

Bulk RNA-sequencing was performed on tumor samples from 33 dogs with either CD4+ (26/33), CD8+ (4/33), or CD4-CD8- (3/33) PTCL as diagnosed by flow cytometry, and sorted CD4+ and CD8+ lymphocytes from healthy control dogs. Following normalization of RNA-seq data, we performed differential gene expression and unsupervised clustering methods. Gene set enrichment analysis was performed to determine the enrichment of canine CD4+ PTCL for human PTCL-NOS, oncogenic pathways, and various stages of T-cell development gene signatures. We utilized gene set variation analysis to evaluate individual canine CD4+ PTCLs for various human and murine T-cell and thymocyte gene signatures. Cultured canine PTCL cells were treated with a pan-PI3K inhibitor, and cell survival and proliferation were compared to DMSO-treated controls. Expression of GATA3 and phosphorylated AKT was validated by immunohistochemistry.

**Results:**

While the canine CD4+ PTCL phenotype exhibited a consistent gene expression profile, the expression profiles of CD8+ and CD4-CD8- canine PTCLs were more heterogeneous. Canine CD4+ PTCL had increased expression of GATA3, upregulation of its target genes, enrichment for PI3K/AKT/mTOR signaling, and downregulation of PTEN, features consistent with the more aggressive GATA3-PTCL subtype of human PTCL-NOS. In vitro assays validated the reliance of canine CD4+ PTCL cells on PI3K/AKT/mTOR signaling for survival and proliferation. Canine CD4+ PTCL was enriched for thymic precursor gene signatures, exhibited increased expression of markers of immaturity (*CD34, KIT*, *DNTT,* and *CCR9*), and downregulated genes associated with the T-cell receptor, MHC class II associated genes (*DLA-DQA1, DLA-DRA, HLA-DQB1,* and *HLA-DQB2*), and CD25.

**Conclusions:**

Canine CD4+ PTCL most closely resembled the GATA3-PTCL subtype of PTCL-NOS and may originate from an earlier stage of T-cell development than the more conventionally posited mature T-helper cell origin.

**Supplementary Information:**

The online version contains supplementary material available at 10.1186/s12885-023-11762-w.

## Introduction

Peripheral T-cell lymphoma (PTCL) refers to a poorly understood heterogeneous group of non-Hodgkin lymphomas arising from a T-lymphocyte cell of origin [1]. While these tumors are diverse in their clinical presentation and histologic and immunophenotypic features, they are similar in their aggressive clinical course, poor responses to treatment, and short survival times [1]. Given their heterogeneity, attempts to subtype PTCLs based on their clinical and immunophenotypic features have been challenging, and approximately one-third of all PTCL cases fall under the “catch-all” diagnosis of PTCL-not otherwise specified (PTCL-NOS), which represents the most common type of PTCL diagnosed globally [2].

Recently, gene expression profiling has helped further divide this ambiguous diagnosis into prognostically significant subtypes—TBX21-PTCL and GATA3-PTCL—with the GATA3-PTCL subtype being associated with lower overall survival times [3]. The TBX21-PTCL subtype is characterized by enrichment for features of T helper 1 (Th1) differentiation of helper T-cells, such as increased expression of IFN-γ and NF-κB, and enrichment for cytotoxic T-cell gene signatures [3]. The GATA3-PTCL subtype, on the other hand, is characterized by strong expression of the transcription factor GATA3 [3], which is essential for T-cell development and for promoting T helper type 2 (Th2) differentiation of helper T-cells [4]; enrichment for cellular proliferation pathways MYC and PI3K/AKT/mTOR [3]; and frequent mutations in the tumor suppressor PTEN [5]. Copy number aberrations affecting genes in the T-cell receptor signaling pathway are commonly observed in both subtypes [5].

In dogs, the most common forms of nodal T-cell lymphoma include PTCL and T-zone lymphoma (TZL) [6]. While TZL in humans is recognized solely as a morphologic variant of PTCL-NOS, TZL in dogs is typically discussed as a distinct entity due to its relatively high prevalence, its unique CD3+ CD5+ CD25+ CD45- immunophenotype, and its indolent clinical course [7]. Non-TZL PTCL in dogs, however, is an aggressive clinical disease with a more variable immunophenotype [8, 9] and median survival times ranging from 97 to 235 days with CHOP or modified CHOP chemotherapy [10, 11].

The clinical, flow cytometric, cytologic, and histologic features of non-TZL PTCL in dogs closely resemble PTCL-NOS [8], and PTCL is relatively more common in dogs than in humans [12]. This has led to their interest as a potential naturally occurring pre-clinical model for this disease. However, similarities between the gene expression profile of human PTCL-NOS and canine PTCL remain largely unknown. Currently, information on the gene expression profile of canine PTCL is limited to a single study of 6 cases of canine PTCL that found expression of CD4 by flow cytometry correlated with a distinctive histomorphology and gene expression profile [8]. While enrichment for the PI3K/AKT/mTOR signaling pathway and downregulation of PTEN was noted in these cases (features which are also observed in the GATA3-PTCL subtype of human PTCL-NOS), this study [8] did not make direct comparisons to human PTCL-NOS and did not report whether other features of the GATA3-PTCL or TBX21-PTCL subtypes of human PTCL-NOS were observed in their cohort.

Additionally, there has been no investigation into the cell of origin of canine PTCL using gene expression data. The cell of origin of human PTCL-NOS remains a subject of ongoing speculation. While the recently identified TBX21-PTCL and GATA3-PTCL subgroups in human PTCL-NOS invited suppositions that these tumors are derived from their normal Th1 and Th2 immune cell counterparts [13], the well-established plasticity of T-helper cell differentiation [14] and the resistance of mature T-cells to oncogenic transformation in mice [15] raises an alternative possibility: that these tumors may arise from an immature precursor that is capable of differentiating into a more mature phenotype. Furthermore, GATA-3 has been shown to function as a proto-oncogene and be expressed in many T-cell tumor types, including precursor neoplasms, independent of the expression of other Th2-specific genes [16]. Investigation into the cell of origin of canine PTCL may offer additional insight into the cell of origin in human PTCL-NOS.

Therefore, the objectives of our study were to 1) determine whether the gene expression profile of canine PTCL is analogous to human PTCL-NOS to further establish the dog as a naturally occurring preclinical model of this disease, and 2) to investigate the cell of origin of canine PTCL by conducting gene set enrichment/variation analyses (GSEA/GSVA) and evaluating the differential expression of various markers of immaturity, T-cell subset markers, and T-cell receptor (TCR)-associated genes.

## Methods

### Sample collection

Fine needle tissue aspirates submitted to the Clinical Hematopathology Lab at Colorado State University for routine diagnostics and identified as PTCL by flow cytometry using the methods and antibody panels described in Seelig et al. [7] were considered for this study. The development and performance of these antibodies in dogs has been previously described [17, 18]. Cases were diagnosed as PTCL by flow cytometry if they exhibited a homogeneous expansion of T-cells, as characterized by the expression of pan-leukocyte antigen CD45, T-cell antigen CD3 (± CD5), and an absence of B-cell antigen CD21 or the stem cell antigen CD34. A previous study from our laboratory demonstrated that 70/70 cases of nodal lymphoma with this phenotype and histologic preparations sufficient for subtyping were consistent with the histologic classification of PTCL [8]. For the current study, a total of 33 samples diagnosed as PTCL by these criteria were selected for RNA-sequencing (RNA-seq) analysis. The site sampled (as reported by the submitting veterinarians) was a peripheral lymph node in 29 cases, mediastinum in 3 cases, and bone marrow in 1 case. Additional patient information is available in Supplementary Table 1A. The selected cases exhibited variable expression of T-cell subset antigens CD4 and CD8 by flow cytometry and were subsequently categorized into CD4+ PTCL (26/33 cases), CD8+ PTCL (4/33 cases), and CD4-CD8- PTCL (3/33 cases). PTCL samples had a median viability of 89% (range 42–98.5%) and a median purity of 91.5% (range 57–98%) (Supplementary Table 1B).

Control T-cells were obtained from the lymph nodes of healthy 1-year-old male (2/9) and female (8/9) dogs designated for IACUC-approved surgical continuing education courses. Healthy control dogs were confirmed to have no evidence of lymphadenopathy, lymphoma, or other morbidities by physical examination, gross necropsy, and flow cytometry of the peripheral lymph nodes. Three of these healthy control dogs were Beagles, 5 were Hounds, and 1 was a mixed breed. Three of the control samples in our study were composed of pooled cells from 2 of the control dogs and the remaining 4 control samples consisted of cells from only one control dog (Supplementary Table 1A). Cells were sorted into CD4+ and CD8+ T-cell subsets by a MoFlo cell sorter (Beckman Coulter, Brea, CA) using positive selection with anti-CD5 (clone YKIX322.3) and anti-CD4 (clone YKIX302.9) or anti-CD8 (clone YCATE55.9). Samples were only utilized if the desired population comprised over 90% of the sorted sample after purity analysis.

### Flow cytometry of normal canine thymocytes

Normal thymocytes were obtained fresh from the thymus of 8 healthy female mixed breed dogs designated for IACUC-approved medical device research. Six of these dogs were 1 year old, 1 dog was 2 years old, and 1 dog was 7 months old (Supplementary Table 1A). Surface CD4, CD8, CD25, and MHC class II expression on fresh thymocytes was evaluated by flow cytometry using the methods as described in Seelig et al. [7] and the flow cytometry antibody panels Panel 2 (for samples CF47 and CF48) and Panel 3 (for samples CF41-CF46) as described in Rout et al. [19].

### RNA-sequencing

RNA from PTCL and control samples was extracted using the Purelink RNA Mini Kit (Thermo Fisher Scientific, Waltham, MA) and quality measured with an Agilent 2100 Bioanalyzer System. The median RIN was 8.45, with a range of 6.3–9.8. RNA samples were delivered to Novogene Corporation Inc (Sacramento, CA) for library construction and sequencing. Sequencing was performed using an Illumina Hiseq PE150 platform. The number of raw reads, clean reads, raw bases, clean bases, and the error rate, phred quality scores, and GC content for each sample was reported by Novogene (Supplementary Table 6A).

### Data analysis

Adapters and low-quality reads were trimmed from raw RNA-seq FASTQ files using fastp (version 0.23.1) [20]. Reads were then aligned to the CanFam 3.1 reference genome (accessed through Ensembl release 104 [21]) with STAR (version 2.7.10a) [22], and the number of reads per gene tabulated by featureCounts (version 2.0.1) [23]. MultiQC (version 1.11) [24] was used to obtain the alignment scores of raw reads. Data normalization using median of ratios, differential expression analysis, and principal component analysis (PCA) was conducted with DESeq2 (version 1.34.0) [25] using default parameters. Low count genes (≤ 10) were pre-filtered prior to running DESeq2 functions. One sample that had a variance in one PCA dimension that was > 3 × the standard deviation of all samples was excluded as an outlier from downstream differential expression analyses and gene set enrichment analyses. Volcano plots of gene expression data were generated with EnhancedVolcano (version 1.12.0) [26]. Scatter plots of normalized count data were generated using ggplot2 (version 3.3.6) [27].

Heatmaps of variance stabilized transformed normalized count data were generated and hierarchical clustering analyses performed using pheatmap (RRID:SCR_016418, version 1.0.12). Unsupervised hierarchical clustering by Euclidean distance with the Ward clustering method was performed on variance stabilized transformed normalized count data from the top 2000 genes with the highest median absolute derivation, and supervised hierarchical clustering by Euclidean distance with the Ward clustering method was performed on variance stabilized transformed normalized count data from the list of genes associated with the GATA3-PTCL and TBX21-PTCL subtypes as defined in Iqbal et al. [3].

GSEA was performed with clusterProfiler (version 4.2.2) [28] and the Broad Institute’s GSEAPreranked tool (version 4.2.3) [29] using the collective gene sets available through the Molecular Signatures Database (MSigDB) [30] and curated gene sets containing the top 250 upregulated and downregulated genes (ranked by log2 fold change) in human CD4 single-positive (SP) infant thymocytes compared to CD4+ T-cells in adult and infant blood obtained from supplementary materials in Helgeland et al. [31]. The Wald test statistic was selected as the ranking metric for the list of differentially expressed genes in canine CD4+ PTCL (as obtained by DESeq2) for its consideration of *p*-value in conjunction with log2 fold change. Default parameters were used for the GSEAPreranked tool. clusterProfiler GSEA parameters were set to perform 10,000 permutations and enforce a *p*-value cutoff of 0.05 following correction using the Benjamini–Hochberg procedure.

The Broad Institute’s GSEAPreranked tool (version 4.2.3) [29] was also used to compare the ranked list of differentially expressed genes in canine CD4+ PTCL to the top 250 up- and downregulated genes in human PTCL-NOS, based on two previous microarray studies (GSE6338 [32] and GSE132550 [33]). The NCBI GEO2R differential gene expression analysis tool [34] was used to obtain the top 250 upregulated and downregulated genes (as ranked by the Wald test statistic) in human PTCL-NOS compared to control human CD4+ lymphocytes from study GSE6338 [32]. Non-normalized RNA-seq data from study GSE132550 was obtained through the NCBI Gene Expression Omnibus [33]. Data normalization and differential gene expression analysis was then performed using DESeq2 (version 1.34.0) [25].

Gene set variation analysis (GSVA) was conducted using GSVA (version 1.46.0) [35] to calculate the enrichment of individual CD4+ PTCL and control CD4+ lymphocyte samples for the expression profiles of various stages of thymocyte development (GSE1460), resting and activated human immune cells (GSE22886), and murine T-helper cell subsets (GSE14308) available from MSigDB, as well as curated gene sets containing the top 250 upregulated and downregulated genes (ranked by log2 fold change) in human CD4 SP infant thymocytes compared to CD4+ T-cells in adult and infant blood obtained from supplementary materials in Helgeland et al. [31]. Variance stabilized transformed read count data from canine CD4+ PTCL samples and control CD4+ lymphocytes (as performed by DESeq2) was used for this analysis. Differential expression analysis to determine significantly enriched gene sets was subsequently performed on GSVA enrichment scores with limma (version 3.54.1) [36] using default parameters. GSVA results were filtered to include only those gene sets found to be significantly differentially expressed (padj < 0.05), and hierarchical Ward-linkage clustering of GSVA enrichment data based on Spearman’s correlation coefficient for samples and the Pearson correlation coefficient for gene sets was performed as recommended in the GSVA vignette (version 1.46.0). Heatmaps of hierarchical clustering of GSVA enrichment data were generated with the heatmap.2 function in gplots (version 3.1.3) [37].

A Tukey’s multiple comparisons test was performed using GraphPad Prism version 8.0 for Windows, GraphPad Software, San Diego, California USA, www.graphpad.com to evaluate differences in surface marker expression (as evaluated by flow cytometry) between canine thymocytes, canine PTCL, and normal canine nodal CD4+ T-cells.

### Immunohistochemistry

Eight canine PTCL cases were selected from routine diagnostic biopsy or necropsy submissions to the Colorado State University Veterinary Diagnostic Laboratories for immunohistochemistry (IHC) for GATA3 or phosphorylated AKT expression. Of these eight cases, a diagnosis of PTCL was confirmed by flow cytometry (6 cases), immunohistochemistry (1 case), or PCR for clonal TCR rearrangement (1 case) [38]. Normal canine lymph node tissue from healthy dogs was used as a control. Heat induced epitope retrieval was performed on a Leica Bond-Max or Leica Bond III IHC stainer using Bond Epitope Retrieval Solution 2 (Bond Epitope Retrieval Solution 2, Leica Biosystems Newcastle Ltd, Newcastle Upon Tyne, United Kingdom) for 30 min. IHC for GATA3 was performed on two canine PTCL cases using the monoclonal anti-mouse GATA3 antibody (clone 1A12-1d9, Invitrogen, Waltham, MA) was applied at a 1:500 dilution. Labeling was performed on an automated staining platform (Bond-Max, Leica Biosystems Newcastle Ltd, Newcastle Upon Tyne, United Kingdom). Fast Red (Fast Red Substrate System, Dako North America Inc., Carpinteria, CA) was used as a chromogen and slides were counterstained with hematoxylin. Negative controls were incubated in diluent consisting of Tris-buffered saline with carrier protein and homologous nonimmune sera. All sequential steps of the immunostaining procedure were performed on negative controls following incubation. Expression of phosphorylated AKT was evaluated in six canine PTCL cases using the rabbit monoclonal phosph-AKT antibody (Ser473, D9E, XP, Cell Signaling Technology, Danvers, MA) at 1:50 dilution with the mouse and rabbit specific HRP/DAB (ABC) detection IHC kit (ab64264, abcam, Cambridge, United Kingdom).

### Proliferation and cell death assays

Primary CD4+ PTCL cells obtained from fine needle aspirations of peripheral lymph nodes of patients with CD4+ PTCL confirmed by the flow cytometry criteria described above were grown under standard conditions (37 °C, 5% carbon dioxide) in RPMI1640 medium supplemented with 10% fetal bovine serum, 0.05 μmol/ml 2-mercaptoethanol (Milipore Sigma, St. Louis MO), 1 × minimum essential medium (MEM), 78 non-essential amino acids (Milipore Sigma, St. Louis MO), 100U/mL penicillin and 100 μg/mL streptomycin (Milipore Sigma, St. Louis MO), 1 × Glutamax (Gibco, Waltham MA), 10 mM Corning HEPES (Media Tech Inc, Manassas VA) and 1 mM sodium pyruvate (Gibco, Waltham MA).

Primary CD4+ PTCL cells were incubated in 96-well round bottom cell culture plates at a concentration of 5 X 10^5^ cells per well. Cells were harvested at 6- or 24-h time points over a total time frame of 48 h of culture. Cell death was measured by annexin V expression and PI measured by flow cytometry. An Annexin V:FITC assay kit (ANNEX100F, Bio Rad, Hercules CA) was used according to manufacturer’s instructions. Briefly, cells were washed in cold PBS and resuspended in binding buffer. Cells were stained with the Annexin V: FITC antibody for 15 min in the dark at room temperature. Following staining, cells were washed with binding buffer and resuspended in binding buffer (0.01 M Hepes (pH 7.4), 0.14 M NaCl, and 2.5 mM CaCl2 solution) and PI. Samples were acquired on a 3-laser Coulter Gallios flow cytometer and analyzed with Kaluza software (Beckman Coulter, Brea, CA). For PI3K inhibition assays, neoplastic T-cells were treated with 50 µM LY294002 (Cell Signaling Technology, Danvers MA) or equivalent volume of DMSO.

Data from these cell culture assays was analyzed in GraphPad Prism version 8.0 for Windows, GraphPad Software, San Diego, California USA, www.graphpad.com. Evaluation of changes in total cell number and CFSE median fluorescence intensity (MFI) over short term culture was performed using multiple t-test analyses to conduct individual comparisons per timepoint. Comparison of total cell death in short term cultures were compared using a Wilcoxon matched pairs signed rank test.

## Results

### Clinical features of canine PTCL

The most represented breeds in our study included mixed breed dogs (8/33, 24%), Boxers (6/33, 18%), and Golden Retrievers (5/33, 15%). Patient ages ranged from 3 to 13 years, with a median age of 6 years. Male dogs were overrepresented at 23/33 cases (70%). This finding is consistent with previous demographic data [8, 39].

The most frequent clinical presentation of canine PTCL in our study was peripheral lymphadenopathy, which was reported in 28/33 cases (85%). 7/33 (21%) had a reported mediastinal mass. Hypercalcemia was reported in 7/33 cases (21%) and hyperglobulinemia in 4 cases (12%). 3 cases (9%) and 1 case (3%) had reported splenomegaly and hepatomegaly, respectively. Other more sporadically reported clinical signs included visceral lymphadenopathy (2/33 cases, 6%), neutrophilia (1/33 cases, 3%), pleural effusion (1/33 cases, 3%), and thrombocytopenia (1/33 cases, 3%). One case had a concurrent cutaneous melanoma diagnosed by cytology, and 1 case had a reported history of blindness and possible brain tumor. Information on bone marrow involvement in this study is limited to 1 case (in which CD4+ PTCL was diagnosed in a sample of bone marrow), as this site is not routinely sampled in dogs.

### CD4 expression by flow cytometry identifies a population of canine T-cell lymphomas with a unique gene expression profile

**Fig. 1:**
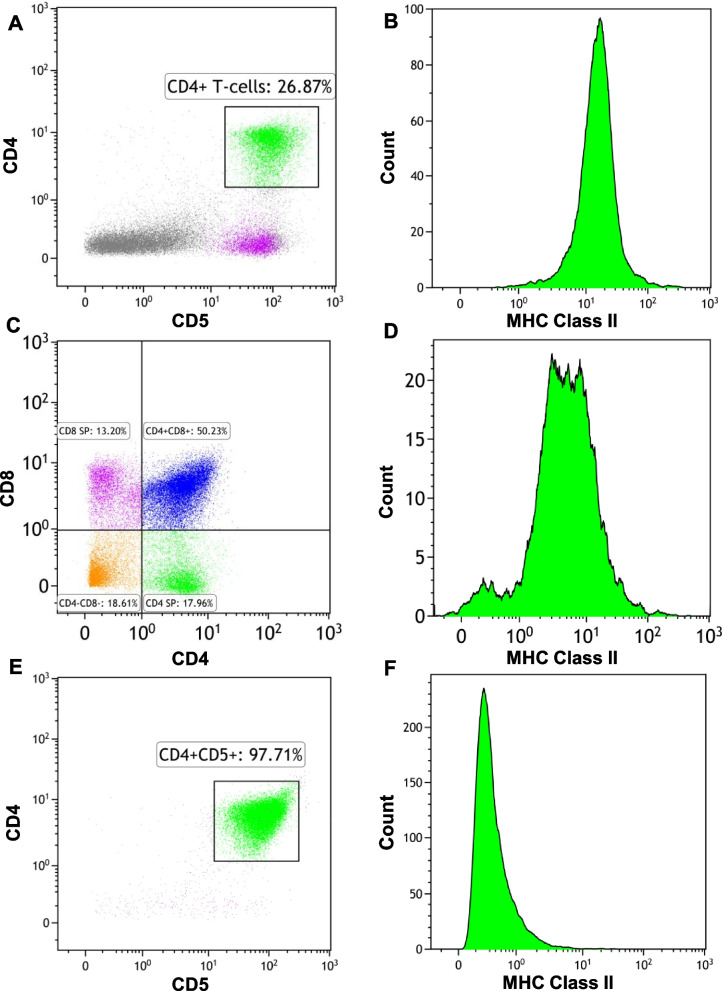
Flow cytometric features of normal mature CD4+ T-cells and normal CD4 SP thymocytes compared to canine CD4+ PTCL cells. **A** Flow cytometry of a normal lymph node from a healthy control dog. CD4+ CD5+ T-cells are highlighted in green. **B** Normal mature nodal CD4+ T-cells exhibit high MHC class II expression. **C** Flow cytometry of normal thymocytes from a healthy control dog. CD4 SP thymocytes are highlighted in green. **D** CD4 SP thymocytes exhibit lower MHC class II expression compared mature nodal CD4+ T-cells. **E** Flow cytometry of canine CD4+ PTCL reveals a homogeneous population of CD4+ CD5+ T-cells. **F** Canine CD4+ PTCL cells exhibit lower MHC class II expression compared to nodal mature T-cells from healthy control dogs

By flow cytometry, cases of canine PTCL varied in their expression of CD4 and CD8, allowing subgrouping into the following most common immunophenotypes: CD4+ PTCL, CD8+ PTCL, and CD4-CD8- PTCL. Among the CD4+ PTCL cases, loss of CD5 expression was observed by flow cytometry in 10/26 (38%) cases. 1 case categorized as CD4+ PTCL demonstrated loss of CD3 expression by flow cytometry. In all but 3 cases, CD25 expression was detected in < 1% of neoplastic cells by flow cytometry (Supplementary Table 1B), and all tumors demonstrated CD45 expression by flow cytometry, ruling out TZL as a potential diagnosis. All immunophenotypes exhibited significantly lower expression of MHC class II by flow cytometry compared to mature CD4+ T-cells from the lymph nodes of healthy control dogs (Fig. 1, Supplementary Fig. 1, Supplementary Table 1B-C).

**Fig. 2:**
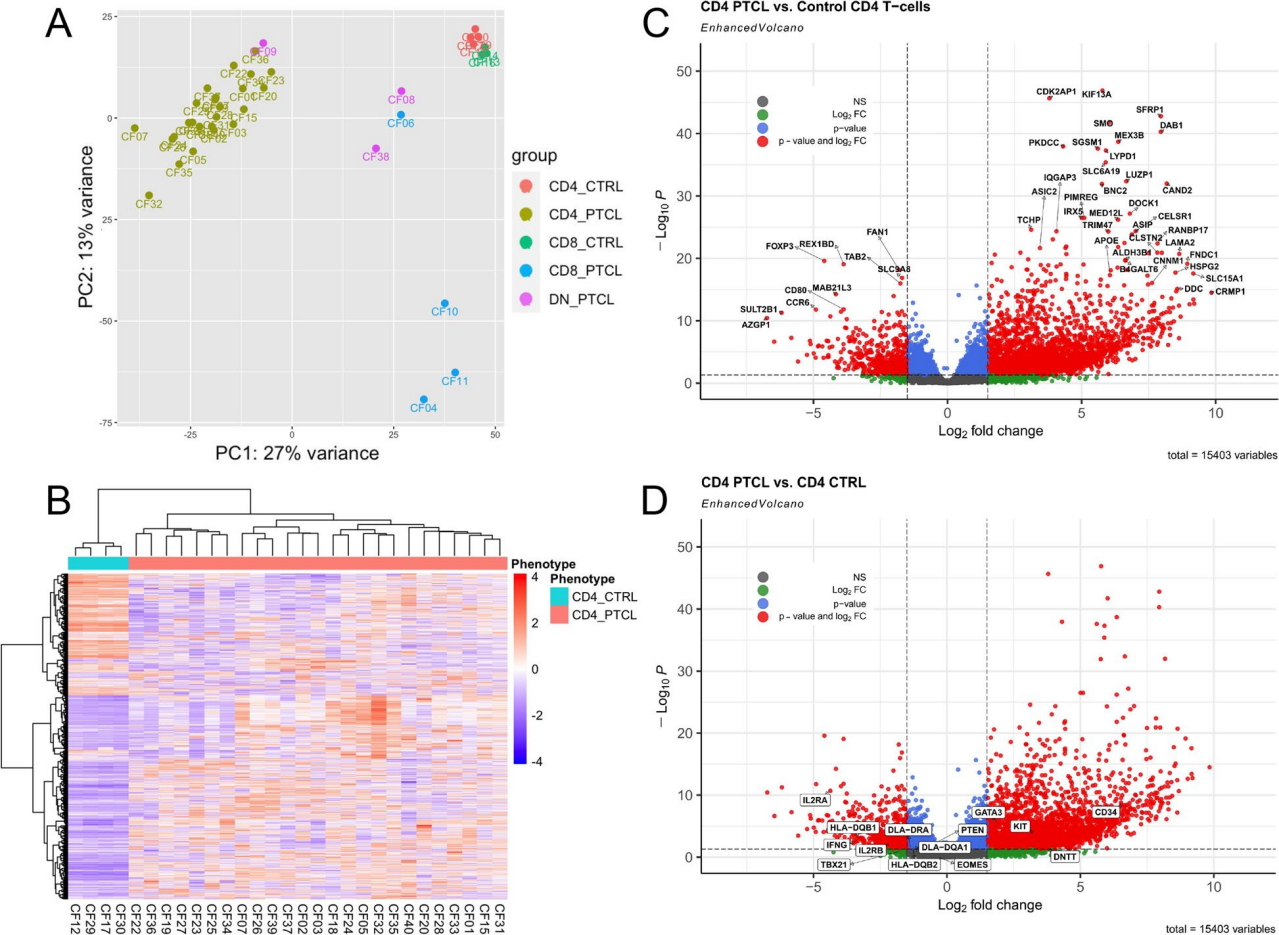
The gene expression profile of canine CD4+ PTCL. **A** Principal component analysis (PCA) of canine PTCL and control samples. Control CD4+ lymphocytes (red) and control CD8+ lymphocytes (green) consistently clustered away from tumor samples. CD4 expression by flow cytometry identified a distinctive subtype of canine PTCL with a uniform gene expression profile (yellow). Wider variance was observed between CD8+ PTCL and CD4-CD8- PTCL samples, suggesting a more heterogeneous gene expression profile among these canine PTCL immunophenotypes. **B** Unsupervised hierarchical clustering of variance stabilized transformed count data from CD4+ PTCL samples and control CD4+ lymphocytes using average Euclidean distance and Ward clustering of the top 2000 genes with the highest median absolute derivation. Upregulated genes are shown in red, while downregulated genes are shown in blue. White represents no change in gene expression. All CD4+ PTCL samples demonstrate a gene expression profile that is distinct from that of the control CD4+ lymphocytes. **C** Volcano plot illustrating the top differentially expressed genes in canine CD4+ PTCL compared to control canine CD4+ lymphocytes. Upregulated and downregulated genes (defined as having a log2 fold change of > 1.5 and < -1.5, respectively) are delineated by the dotted vertical lines, and the red data points indicate those genes that were significantly upregulated or downregulated (based on a Benjamini–Hochberg adjusted *p*-value cutoff of 0.05). Blue data points indicate those genes that met our cutoff for significance but had a log2 fold change with an absolute value less than 1.5. Green data points indicate those genes that met our cutoff for log2 fold change but were not statistically significant. Gray data points indicate those genes that met neither our log2 fold change nor our *p*-value cutoff. **D** Volcano plot illustrating the differential expression of selected genes of interest in canine CD4+ PTCL compared to control CD4+ lymphocytes, including Th1- and Th2-associated genes, markers of immaturity, MHC class II-associated genes, and genes associated with T-cell activation

Initial principal component analysis (PCA) of differential gene expression data revealed one outlier case (Supplementary Fig. 2) in the CD4+ PTCL group that was subsequently excluded from subsequent PCA and downstream analyses. Notably, this case had high expression of CD30 (normalized count = 8471.48, mean = 358.98, standard deviation = 1622.66), a feature of anaplastic large cell lymphoma [40], which may have accounted for its clustering away from the typical canine CD4+ PTCL cases. PCA revealed consistent clustering of control canine CD4 and CD8 lymphocytes away from tumor samples, and close clustering of canine CD4+ PTCLs (Fig. 2A), corroborating previous findings that CD4 expression by flow cytometry corresponds to a distinct subgroup of canine PTCL with a uniform gene expression profile [8]. With one exception, the CD8+ and CD4-CD8- tumors did not cluster with the CD4+ lymphomas and exhibited more heterogeneity, suggesting these tumor types are from more than one lineage, and from a different lineage than CD4+ PTCL (Fig. 2A). We elected to focus on the distinctive canine CD4+ PTCL immunophenotype for further differential gene expression and gene set enrichment analyses.

### Canine CD4+ PTCL consistently exhibits a gene expression profile that is distinct from that of control CD4+ lymphocytes

RNA-seq data quality parameters are summarized in Supplementary Table 6A and 6B. The median number of raw reads per sample was 58,089,233 (range: 46,655,240–81,854,562 reads). Error rates ranged from 0.02% to 0.03%. The average GC content was 50.88%. The average percentage of reads able to be uniquely mapped to the reference genome per sample was 93.74%, and the average percentage of reads that were found to overlap known genes was 61.07%.

Unsupervised hierarchical clustering of variance stabilized transformed count data from CD4+ PTCL samples and control CD4+ lymphocytes using average Euclidean distance and Ward clustering of the top 2000 genes with the highest median absolute derivation revealed a consistent gene expression profile across all control canine CD4+ lymphocyte samples from healthy dogs (Fig. 2B). All canine CD4+ PTCL samples demonstrated a more heterogeneous gene expression profile that was distinct from that of control CD4+ lymphocytes (Fig. 2B). 38,456 genes and 15,816 genes were found to be significantly up- and downregulated, respectively, in canine CD4+ PTCL compared to control CD4+ lymphocytes based on a *p*-value cutoff of 0.05 following Benjamini–Hochberg adjustment (Fig. 2C). The 250 most significantly upregulated genes and 250 most significantly downregulated genes (as ranked by the Wald test statistic) are provided in Supplementary Table 2A. Read counts normalized by median of ratios (as performed by DESeq2) are provided in Supplementary Table 2B.

There was significantly increased expression of transcription factor *GATA3* and markers of immaturity *KIT, CD34*, and *DNTT* (Fig. 2D, Table 1). Expression of *PTEN* and *EOMES* were slightly decreased, but these results were not significant (Fig. 2D, Table 1). There was significantly decreased expression of transcription factor *TBX21*, *IFN-y*, *IL2RA*, and MHC class II associated genes *DLA-DQA1, DLA-DRA, HLA-DQB1*, and *HLA-DQB2* (Fig. 2D, Table 1).

**Table 1:** Log2 fold change and Benjamini–Hochberg adjusted *p*-values of the differentially expressed genes in canine CD4+ PTCL compared to control canine CD4+ lymphocytes labeled in the volcano plot in Fig. 2D

Gene	Log2 fold change	padj
*CD34*	6.527449673	1.63 × 10^–09^
*DNTT*	3.853356134	0.025041122
*KIT*	3.259621943	0.000246273
*GATA3*	1.972010229	5.20 × 10^–06^
*EOMES*	-0.323304866	0.983983004
*PTEN*	-0.735623799	0.026796328
*DLA-DQA1*	-1.217342889	0.042038364
*DLA-DRA*	-1.368224639	0.030936953
*TBX21*	-1.750313162	0.107412113
*IL2RB*	-2.18200111	0.005813881
*HLA-DQB1*	-3.230290232	0.000735723
*HLA-DQB2*	-1.266726858	0.045314148
*IFN-y*	-3.41679866	0.002444061
*IL2RA*	-4.36853914	2.01 × 10^–11^

By GSEA, the top enriched MSigDB gene sets in canine CD4+ PTCL (compared to control canine CD4+ T-cells) included various cancer gene signatures; miscellaneous neural cell, T cell, and NK cell gene signatures; and several cell cycle associated gene neighborhoods (Supplementary Table 3A and 4A).

### Canine CD4+ PTCL exhibits a molecular signature similar to the GATA3-PTCL subtype of PTCL-NOS in humans

**Fig. 3:**
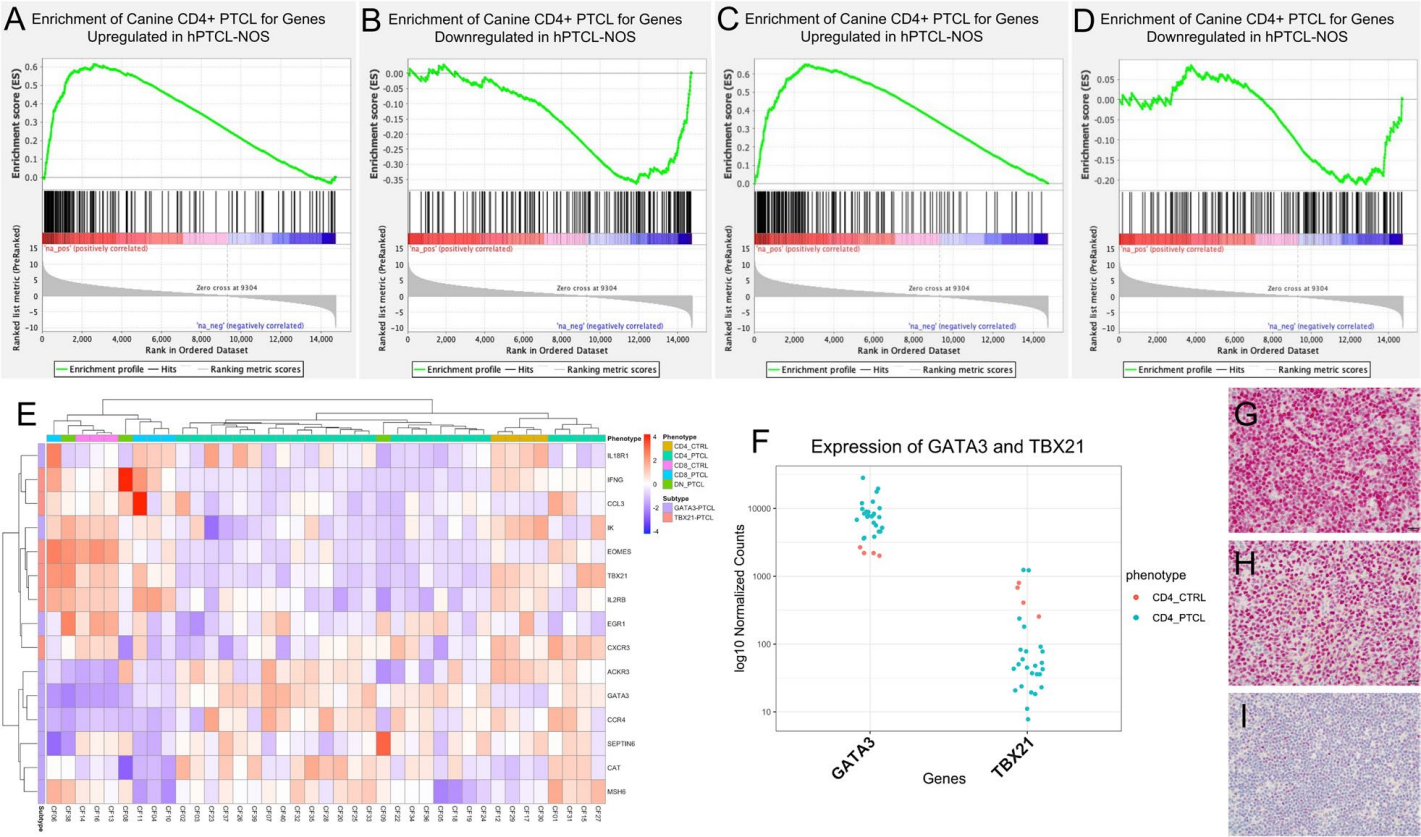
The gene expression profile of canine CD4+ PTCL more closely resembles the GATA3-PTCL subgroup of human PTCL-NOS than the TBX21-PTCL subgroup. **A**-**D** GSEA enrichment plots demonstrating the significant enrichment of the ranked list of differentially expressed genes in canine CD4+ PTCL for the top 250 upregulated genes in human PTCL-NOS (**A**, **C**) and negative enrichment for the 250 most downregulated genes in human PTCL-NOS (**B**, **D**), as obtained from GSE6338 and GSE132550. **E** Hierarchical clustering of canine PTCL samples and control canine CD4+ and CD8+ lymphocytes based on expression levels of genes associated with the human GATA3-PTCL and TBX21-PTCL subtypes of PTCL-NOS (from Iqbal et al. [3]). Hierarchical clustering was done using average Euclidean distance and Ward clustering of variance stabilized transformed normalized RNA-seq count data. Upregulated genes are shown in red, while downregulated genes are shown in blue. The majority of canine CD4+ PTCL cases show preferential expression of the genes associated with the GATA3-PTCL subtype, while other canine PTCL phenotypes are more heterogeneous. **F** A scatter plot demonstrating the differential expression of transcription factors GATA3 (log2 fold change = 1.97, padj = 5.20 × 10^–06^) and TBX21 (log2 fold change = -1.75, padj = 0.1) in canine CD4+ PTCL samples compared to control canine CD4+ lymphocytes. **G**-**I** Canine CD4+ PTCL samples (*n* = 6) demonstrated strong, diffuse nuclear immunostaining for GATA3 by immunohistochemistry (**G**, **H**) compared to sections of control canine lymph nodes (**I**)

GSEA using the ranked list of differentially expressed genes in canine CD4+ PTCL compared to control canine CD4+ lymphocytes revealed significant enrichment of canine CD4+ PTCL for the top 250 upregulated genes in human PTCL-NOS derived from Piccaluga et al. [32] and Etebari et al., and negative enrichment for the 250 most downregulated genes in human PTCL-NOS, although the latter was only significant in the gene set derived from Piccaluga et al. (Table 2, Fig. 3A-D).

**Table 2:** Enrichment scores for canine CD4+ PTCL compared to human PTCL-NOS gene signatures

Gene Set	Normalized enrichment score	*p*-value	FDR
GSE6338: Top 250 upregulated genes in human PTCL-unspecified compared to control CD4+ T-cells	2.31	< 0.001	< 0.001
GSE6338: Top 250 downregulated genes in human PTCL-unspecified compared to control CD4+ T-cells	-1.98	< 0.001	< 0.001
GSE132550: Top 250 upregulated genes in human PTCL-NOS compared to control CD4+ T-cells	2.40	< 0.001	< 0.001
GSE132550: Top 250 downregulated genes in human PTCL-NOS compared to control CD4+ T-cells	-1.16	0.1	0.08

Canine CD4+ PTCL demonstrated increased expression of the genes associated with the GATA3-PTCL subtype and decreased expression of the genes associated with TBX21-PTCL subtype of human PTCL-NOS, as defined by Iqbal et al. (Fig. 3E). Compared to control canine CD4+ lymphocytes, canine CD4+ PTCL had significantly increased expression of GATA3 and decreased expression of TBX21 (Fig. 3F), although the latter was not statistically significant (Table 1). Canine CD4+ PTCL samples also exhibited strong, diffuse immunostaining for GATA3 by immunohistochemistry compared to controls (Fig. 3G-I).

**Fig. 4:**
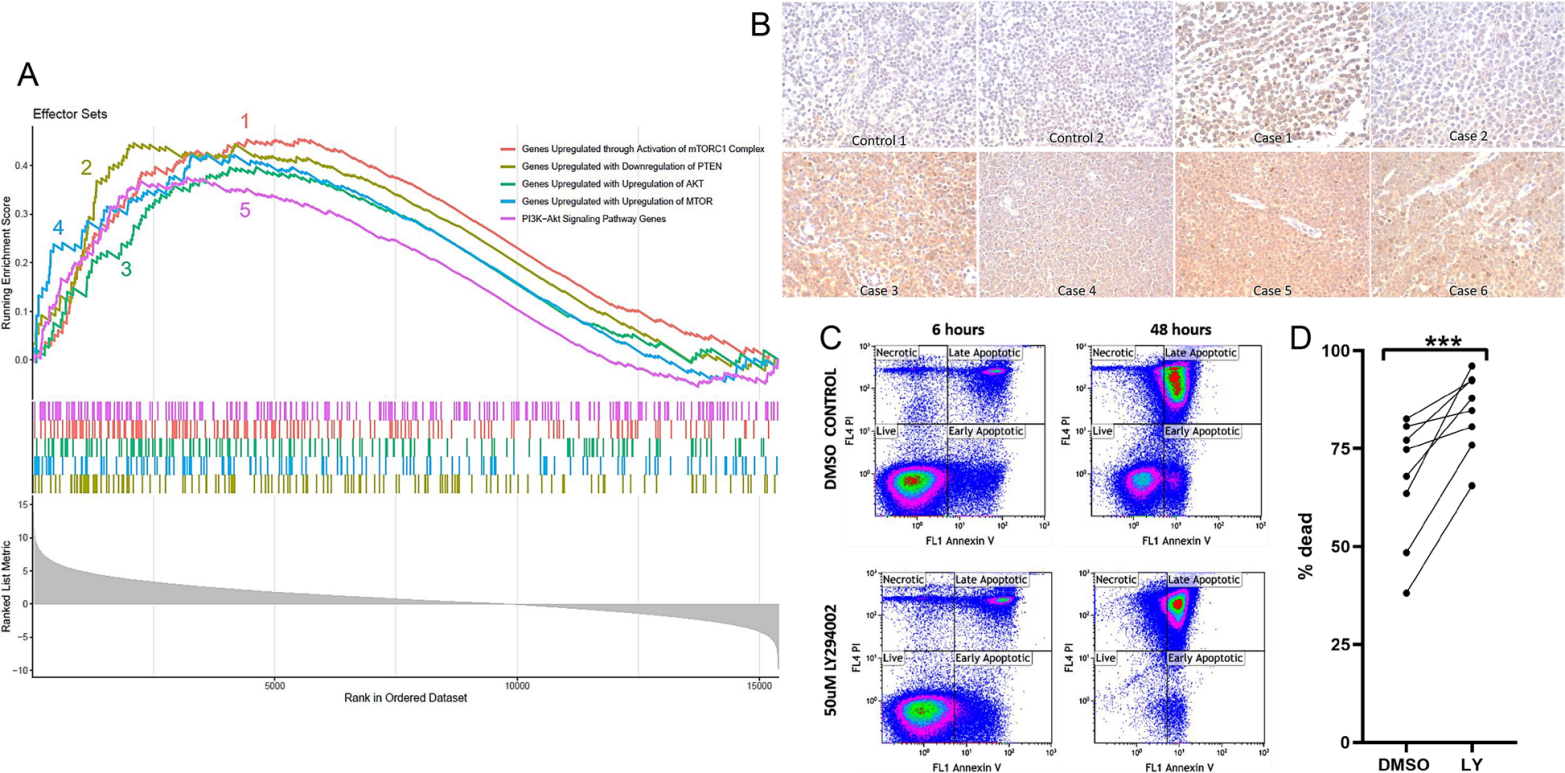
Canine CD4+ PTCL depends on the PI3K/Akt/mTOR signaling pathway for cell survival and proliferation. **A** GSEA enrichment plot demonstrating the significant enrichment (with a Benjamini–Hochberg *p*-value cutoff of 0.05) of the gene expression profile of canine CD4+ PTCL for gene signatures associated with downregulation of PTEN and upregulation of the PI3K/AKT/mTOR signaling cascade. 1 (red) = Enrichment of canine CD4+ PTCL for the gene set upregulated through activation of the mTORC1 complex (hallmark MTORC1 signaling pathway); 2 (yellow) = Enrichment of canine CD4+ PTCL for the gene set upregulated with downregulation of PTEN (GSE7562); 3 (green) = Enrichment of canine CD4+ PTCL for the gene set upregulated with upregulation of AKT (GSE1413); 4 (blue) = Enrichment of canine CD4+ PTCL for the gene set upregulated with upregulation of MTOR (GSE1413); 5 (pink) = Enrichment of canine CD4+ PTCL for PI3K-Akt signaling pathway genes (Wikipathways WP4172). **B** Canine CD4+ PTCL samples (*n* = 6) were diffusely and strongly positive for cytoplasmic expression of phosphorylated AKT by immunohistochemistry compared to sections of control canine lymph nodes. **C**, **D** Primary CD4+ PTCL cells were treated with pan-PI3K inhibitor LY249002 or DMSO control and grown in culture over 48 h. Cellular death was evaluated via annexin V and PI, measured by flow cytometry. A representative flow cytometry plot from one patient (**C**) and a summary of 8 total CD4+ primary patient samples (**D**) illustrate significantly increased tumor cell death following treatment with LY294002

By GSEA, canine CD4+ PTCL was also significantly enriched for gene signatures associated with hallmark PI3K/AKT (WP4172 [41], normalized enrichment score (NES) = 1.42, padj = 0.01, FDR q-value = 0.009) and MTORC1 signaling [30] (NES = 1.64, padj = 0.003, FDR q-value = 0.002), upregulation of AKT (GSE1413 [42], NES = 1.48, padj = 0.04, FDR q-value = 0.03) and mTOR (GSE1413 [42], NES = 1.53, padj = 0.01, FDR q-value = 0.01), and downregulation of PTEN (GSE7562 [43], NES = 1.57, padj = 0.007, FDR q-value = 0.006) (Fig. 4A). Enrichment for PI3K/AKT/mTOR signaling and loss of PTEN are features described in the GATA3-PTCL subtype of human PTCL-NOS [3, 5]. This enrichment for PI3K/AKT/mTOR signaling in our study was validated with immunohistochemistry for phosphorylated AKT and in vitro cell culture assays evaluating tumor cell survival and proliferation following treatment with a PI3K inhibitor. Canine CD4+ PTCL lymph node samples (*n* = 6) exhibited diffuse immunostaining of phosphorylated AKT compared to control lymph nodes by immunohistochemistry (Fig. 4B). Following treatment with LY249002, a pan-PI3K inhibitor, primary canine CD4+ PTCL cells harvested from patient lymph nodes exhibited significantly increased total cell death compared to DMSO-treated controls (*p*-value = 0.0078) (Fig. 4C, D).

### The gene expression profile of canine CD4+ PTCL suggests a thymic cell of origin

**Fig. 5:**
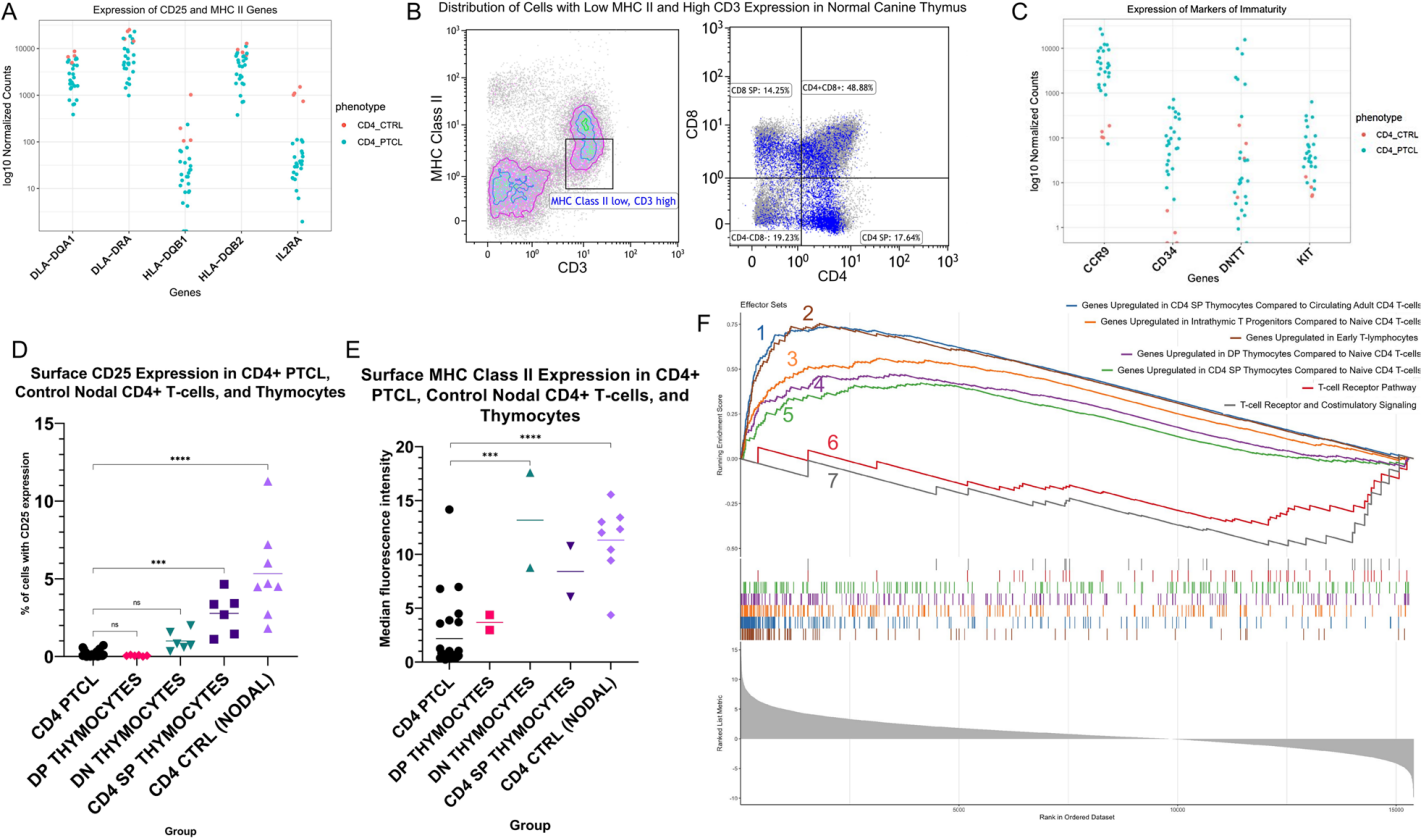
Gene expression profiling of canine CD4+ PTCL suggests a thymic precursor cell of origin. **A**, **C** *IL2RA* and MHC class II genes *DLA-DQA1*, *DLA-DRA*, *HLA-DQB1*, and *HLA-DQB2* were significantly downregulated (**A**) and markers of immaturity *CD34*, *KIT*, *DNTT,* and *CCR9* were significantly upregulated (**C**) in canine CD4+ PTCL compared to control CD4+ T-cells. **B** Distribution of cells with high surface CD3 expression and low surface MHC Class II expression (similar to canine CD4+ PTCL) in a normal canine thymus. While represented throughout all stages of thymocyte development, these cells were most prevalent in DP and CD4 SP thymocytes. **D**, **E** Canine CD4+ PTCL cells exhibited significantly lower surface CD25 expression by flow cytometry compared to normal nodal CD4+ T-cells (padj < 0.0001) and CD4 SP thymocytes (padj = 0.0003), similar surface CD25 expression as DP and DN thymocytes, and significantly lower surface MHC Class II expression compared to normal nodal CD4+ T-cells (*p* < 0.0001) and DN thymocytes (*p* = 0.0007) based on a Tukey’s multiple comparison’s test. **F** 1 (red) = Enrichment of canine CD4+ PTCL for genes upregulated in CD4 SP thymocytes compared to circulating adult CD4+ T-cells (GSE139242); 2 (blue) = Enrichment of canine CD4+ PTCL for genes upregulated in CD4 SP thymocytes compared to circulating infant CD4+ T-cells (GSE139242); 3 (green) = Enrichment of canine CD4+ PTCL for genes upregulated in CD4 SP thymocytes compared to naïve CD4 T-cells (GSE1460); 4 (purple) = Enrichment of canine CD4+ PTCL for genes upregulated in early T-lymphocytes (GSE1460); 5 (orange) = Enrichment of canine CD4+ PTCL for genes upregulated in intrathymic T progenitors compared to naïve CD4 T-cells (GSE1460); 6 (brown) = Negative enrichment of canine CD4+ PTCL for TCR and costimulatory signaling (Wikipathways WP2583); 7 (gray) = Negative enrichment of canine CD4+ PTCL for TCR signaling (Biocarta TCR signaling pathway). Results had a Benjamini–Hochberg *p*-value ≤ 0.05

By flow cytometry, canine CD4+ PTCL samples were noted to have significantly lower CD25 (*p* < 0.0001) (Fig. 5A, D) and MHC Class II expression (*p* < 0.0001) (Figs. 1B, D and 5E) than CD4+ T-cells from control lymph nodes. Similarly low surface expression of CD25 and MHC Class II was appreciated in normal canine thymocytes by flow cytometry (Figs. 1E and 5D, E), suggesting that low expression of these surface antigens may be reflective of earlier stages of T-cell maturation. Specifically, concurrent high surface CD3 expression with low surface MHC class II expression, like what was observed in canine CD4+ PTCL cells, was most characteristic of DP and CD4 SP thymocytes in the canine thymus (Fig. 5B). The low CD25 and MHC class II surface expression in canine CD4+ PTCL observed by flow cytometry was corroborated by our gene expression data, which found that canine CD4+ PTCL had significantly decreased expression of *IL2RA* and MHC class II-associated genes compared to control canine CD4+ lymphocytes (Fig. 5A). GSEA comparing the gene expression profile of canine CD4+ PTCL to the Biocarta [44] TCR signaling pathway and Wikipathways [41] TCR and co-stimulatory signaling pathway (WP2583) found that canine CD4+ PTCL was negatively enriched for these gene signatures (NES = -1.58, FDR q-value = 0.01; and NES = -1.80, FDR q-value = 0.03, respectively) (Fig. 5F), indicating that TCR signaling may be downregulated in canine CD4+ PTCL. Taken together, these findings suggest that canine CD4+ PTCL does not originate from an activated CD4+ T-cell phenotype.

GSEA comparing the gene expression profile of canine CD4+ PTCL to gene sets associated with subpopulations of human thymocytes and circulating naïve CD4+ T-cells (GSE1460 [45] and GSE139242 [31]) revealed significant enrichment of canine CD4+ PTCL for gene signatures associated with early T lymphocytes, intrathymic T progenitor cells, CD4 SP thymocytes, and CD4/CD8 double-positive (DP) thymocytes compared to naïve circulating CD4+ T-cells (Table 3, Fig. 5F).

**Table 3:** Enrichment scores for canine CD4+ PTCL compared to human thymocyte and early T-cell gene signatures

Gene Set	Normalized enrichment score	padj	FDR
GSE1460: Early T lymphocyte gene signature	2.66	0.003	0.002
GSE1460: Genes upregulated in intrathymic T-progenitor cells compared to naïve CD4+ T-cells	2.08	0.003	0.002
GSE1460: Genes upregulated in CD4 SP thymocytes compared to naïve CD4+ T-cells	1.58	0.006	0.005
GSE1460: Genes upregulated in CD4/CD8 DP thymocytes compared to naïve CD4+ T-cells	1.51	0.01	0.01
GSE139242: Genes upregulated in infant CD4+ thymocytes compared to CD4+ T-cells in infant blood	2.78	4.25e-10	5.26e-11
GSE139242: Genes upregulated in infant CD4+ thymocytes compared to CD4+ T-cells in adult blood	2.50	4.25e-10	5.26e-11

Additionally, differential gene expression analyses revealed significantly increased expression of markers of immaturity and genes expressed during development, such as *CD34* (log2 fold change = 6.53, padj = 1.63 × 10^–09^), *KIT* (log2 fold change = 3.26, padj = 0.0002), *DNTT* (log2 fold change = 3.85, padj = 0.03), and *CCR9* (log2 fold change = 5.27, padj = 6.67 × 10^–12^) in canine CD4+ PTCL compared to control canine CD4+ lymphocytes (Fig. 5C). Although *CD34* message is detected, protein expression of CD34 on these cells is consistently negative.

GSEA comparing the gene expression profile of canine CD4+ PTCL to gene sets associated with various mature murine T-cell lineages and naïve CD4+ T-cells (GSE14308) found no significant enrichment for Th1, Th2, Th17, or Treg signatures compared to naïve CD4+ T-cells in canine CD4+ PTCL (Supplementary Table 3A and 4A). Of note, there was one contradictory gene expression study (GSE22886) that showed negative enrichment of canine CD4+ PTCL for genes upregulated in naïve CD4+ T-cells compared to activated Th1 and Th2 cells (Supplementary Table 3A and 4A).

**Fig. 6:**
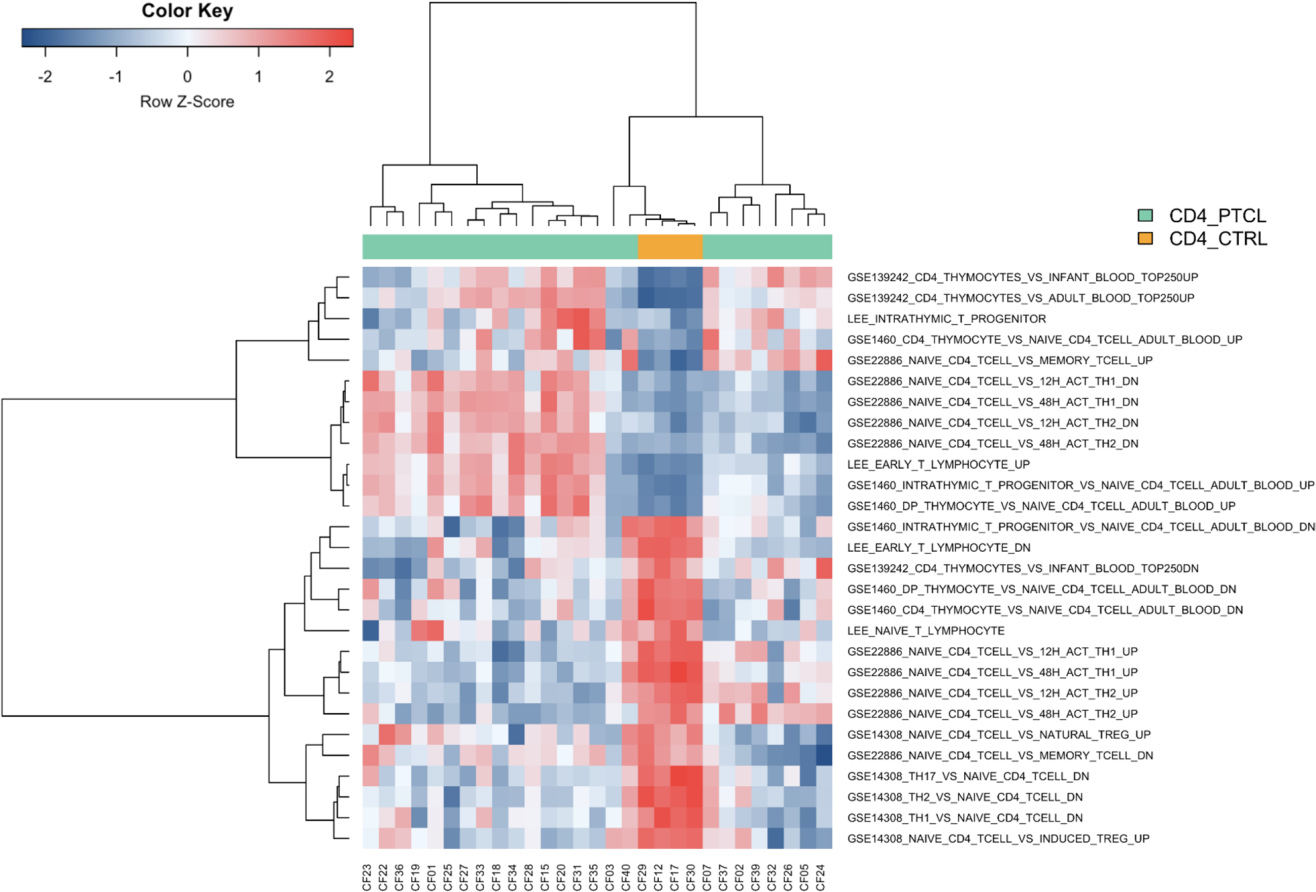
GSVA evaluating the enrichment of individual samples for the expression profiles of various stages of thymocyte development (GSE1460), resting and activated human T cells (GSE22886), murine T-helper cell subsets (GSE14308), and the top up- and downregulated genes in human CD4 SP thymocytes compared to CD4+ T-cells in adult and infant blood (GSE139242). Control canine CD4+ lymphocytes are uniformly negatively enriched for early T-cell progenitor and thymocyte gene signatures and strongly enriched for naïve T-cell signatures. Canine CD4+ PTCL separates into two distinctive clusters, with the first group exhibiting strong enrichment for early T lymphocyte gene signatures, intrathymic T progenitor cells, genes upregulated in DP thymocytes compared to naïve circulating CD4+ T-cells, and genes upregulated with Th1 and Th2 cell activation; as well as variable enrichment for CD4 SP thymocyte gene signatures. Members of the second group are largely negatively enriched or non-enriched for early T lymphocyte and intrathymic T progenitor cell signatures (but still exhibit positive enrichment for CD4 SP thymocyte gene signatures compared to mature circulating CD4+ T-cells) and are positively enriched for genes upregulated in naïve CD4+ T-cells compared to activated Th1 and Th2 cells

Enrichment of individual canine CD4+ PTCL samples and control canine CD4+ lymphocyte samples for the expression profiles of various stages of thymocyte development (GSE1460), resting and activated human immune cells (GSE22886), murine T-helper cell subsets (GSE14308), and the top 250 upregulated and downregulated genes in human CD4 SP infant thymocytes compared to CD4+ T-cells in adult and infant blood (from Helgeland et al. [31]) was evaluated by GSVA. Control samples were negatively enriched for early T-cell progenitor and thymocyte gene signatures and strongly enriched for naïve T-cell signatures (Supplementary Table 5, Fig. 6). Two distinct clusters were identified within the CD4+ PTCL phenotype by unsupervised hierarchical clustering of GSVA scores (Fig. 6). The first group was characterized by strong enrichment of tumor cells for early T lymphocyte gene signatures, intrathymic T progenitor cells, genes upregulated in DP thymocytes compared to naïve circulating CD4+ T-cells, and genes downregulated in naïve CD4+ T-cells compared to activated Th1 and Th2 cells. Members of this group were variably enriched for CD4 SP thymocyte gene signatures and often either negatively enriched or non-enriched for genes upregulated in naïve CD4+ T-cells compared to activated Th1 and Th2 cells (Supplementary Table 5, Fig. 6). Members of the second group observed by hierarchical clustering of GSVA within the CD4+ PTCL phenotype were largely negatively enriched or non-enriched for early T lymphocyte gene signatures, intrathymic T progenitor cells, and genes upregulated in DP thymocytes compared to naïve circulating CD4+ T-cells. Instead, this group showed enrichment for CD4 SP thymocyte gene signatures and genes upregulated in naïve CD4+ T-cells compared to activated Th1 and Th2 cells (Supplementary Table 5, Fig. 6). This distinctive clustering within our canine CD4+ PTCL cases may suggest the existence of subtypes of canine CD4+ PTCL with varying degrees of T-cell differentiation.

## Discussion

In this study, we investigated the gene expression profile of canine PTCL to determine whether canine PTCL resembles human PTCL-NOS and to investigate the cell of origin of canine PTCL. We found that the gene expression profile of canine CD4+ PTCL resembles the human GATA3 PTCL-NOS subtype and in both species, these PTCL are enriched for the PI3K/AKT/MTOR pathway. Further, gene and antigen expression in our study favored a thymic precursor cell of origin over a more mature or activated T-cell phenotype.

The immunophenotype most commonly diagnosed by flow cytometry in dogs, CD4+ PTCL, exhibited the most consistent gene expression profile, whereas the gene expression profiles of CD8+ and CD4-CD8- canine PTCLs suggested a different cell of origin. This observed heterogeneity may be due in part to the small sample sizes representing these phenotypes. We elected to focus on 25 cases of the distinct CD4+ PTCL immunophenotype for our study.

Our study revealed several similarities between canine PTCL and human PTCL-NOS, including similarities in clinical presentation, immunophenotype, and gene expression. In humans, a nodal presentation is most common with PTCL-NOS [2], and peripheral lymphadenopathy was the most frequently reported clinical presentation of canine PTCL in our study (85%). In humans, there is a reported male predominance of 1.5–1.9:1 [2], and male dogs were similarly overrepresented in our study (70%). Hypercalcemia is a commonly observed feature on biochemistry profiles in human PTCL-NOS [2], and 21% of canine PTCL cases in our study were hypercalcemic. One difference between human and canine PTCL is frequent mediastinal involvement of the canine tumor.

By flow cytometry, canine CD4+ PTCLs expressed the pan-leukocyte marker CD45 and T-cell marker CD3, but loss of CD5 was observed in 38% of cases. Similarly, the majority of PTCL-NOS cases in humans lose expression of one or more mature T-cell antigens, most often CD7 or CD5 [2].

GSEA revealed significant enrichment of canine CD4+ PTCL for the top 250 upregulated genes in human PTCL-NOS and significant negative enrichment for the top 250 downregulated genes in human PTCL-NOS based on a previous microarray study [32], suggesting that canine CD4+ PTCL and human PTCL-NOS are comparable diseases at a gene expression level.

Of the two recently described molecular subgroups of human PTCL-NOS, TBX21-PTCL and GATA3-PTCL, the gene expression profile of canine CD4+ PTCL most closely resembled that of the GATA3-PTCL subtype. In humans, this subtype is associated with a poorer prognosis and characterized by increased expression of transcription factor GATA3 and its target genes [3]. Similarly, canine CD4+ PTCL exhibited increased expression of *GATA3* and decreased expression of *TBX21*, although the latter was not statistically significant. Increased expression of GATA3 was also demonstrated in peripheral nodes of canine CD4+ PTCL samples compared to control lymph nodes from healthy dogs via immunohistochemistry. Canine CD4+ PTCL also tended to upregulate other genes associated with the GATA3-PTCL subtype (such as *IL18R1*, *CCR4*, *CAT*, and *ACKR3*) and downregulate other genes associated with the TBX21-PTCL subtype (such as *IFNG*, *IL2RB*, *EOMES*, and *CCL3*) compared to other canine PTCL phenotypes and control canine CD4+ and CD8+ lymphocytes.

In humans, the GATA3-PTCL subtype is also characterized by enrichment for the MYC and PI3K/AKT/mTOR signaling pathways that promote cellular proliferation [3], and frequent mutations in the tumor suppressor gene *PTEN* [5]. GSEA revealed significant enrichment of the gene expression profile of canine CD4+ PTCL for gene signatures associated with upregulation of the PI3K/AKT/mTOR pathway and downregulation of PTEN. This corroborates previous studies that found PTEN-mTOR pathway mutations in almost half of “Boxer type” canine T-cell lymphomas [46], which are most likely the CD4+ PTCL group being examined here. Diffuse expression of phosphorylated AKT in the lymph nodes of canine PTCL samples compared to control lymph nodes from healthy dogs was also demonstrated with immunohistochemistry. Inhibition of PI3K in vitro resulted in decreased proliferation and increased cell death in primary CD4+ PTCL cells harvested from patient lymph nodes, further illustrating the reliance of canine CD4+ PTCL on PI3K/AKT/mTOR signaling for tumor cell proliferation and survival. These findings further support a similar molecular pathogenesis between the GATA3-PTCL subtype of human PTCL-NOS and canine CD4+ PTCL. Given the current heterogeneity of clinical and molecular features in human PTCL-NOS, this narrowing down of the most specific subgroup resembled by canine CD4+ PTCL should allow for more precise preclinical applications of our proposed canine model.

While we have demonstrated a similar dependence on PI3K/AKT signaling between human GATA3-PTCL and canine CD4+ PTCL, future studies are warranted to clarify the mechanism and specific downstream effectors implicated in this dependence. The PI3K/AKT/mTOR axis is known to promote cell survival and proliferation through a variety of mechanisms, including through the inhibition of pro-apoptotic proteins like BAD and FOXO [47, 48] and increased synthesis of cyclins [49, 50], among others. Notably, *FOXO1* was downregulated in our neoplastic CD4+ PTCL cells compared to control nodal CD4+ T cells (log2 fold change = -1.2, padj = 0.01). PI3K/AKT signaling also promotes the activity of glycolytic enzymes [51, 52] and the upregulation of glucose transporters [53, 54], which helps produce the cellular metabolic shift toward aerobic glycolysis observed in many cancer cells [55]. One such glucose transporter influenced by PI3K/AKT signaling is GLUT4 [53], encoded by the *SLC2A4* gene. In our study, this gene was upregulated in canine CD4+ PTCL cells compared to control nodal CD4+ T cells (log2 fold change = 2.9, padj = 0.01). For these reasons, we suspect the dependency of CD4+ PTCL cells on this signaling cascade is multifactorial, although additional studies are needed. In human PTCL-NOS, much of the effort to understand the therapeutic applications of the PI3K/AKT/mTOR dependency has been focused on more upstream targets, like utilizing PI3K inhibitors [56], and to our knowledge investigations into more specific downstream effectors remain limited. The correlation between GATA3 upregulation and enrichment for PI3K/AKT/mTOR signaling both in human GATA3-PTCL and canine CD4+ PTCL could reflect a molecular connection between these factors, a finding suggested by a previous study noting that GATA3 target genes in both immature and mature T-cell neoplasms consistently included genes involved in PI3K/AKT signaling [16]. Additionally, as previously mentioned, deletions involving the PI3K inhibitor PTEN are more prevalent in the GATA3-PTCL subtype of human PTCL-NOS than the TBX21-PTCL subtype [5], suggesting that the PI3K/AKT/mTOR enrichment frequently seen in tumors of this subtype may result from an increased susceptibility of this subtype to deletions in *PTEN*. This correlation seems similarly probable in canine CD4+ PTCL based on whole exome sequencing data identifying PTEN-mTOR pathway mutations in almost half of “Boxer type” canine T-cell lymphomas [46]. However, the relationship (if any) between GATA3 upregulation and this increased susceptibility to *PTEN* deletions remains to be explored.

Gene expression profiling of canine CD4+ PTCL also provided insight into a possible cell of origin of these tumors. The cell of origin of human PTCL-NOS remains nebulous, as previous assumptions that the recently identified TBX21-PTCL and GATA3-PTCL subtypes were derived from their normal Th1 and Th2 immune cell counterparts have been more recently met with cautious skepticism given the demonstrable plasticity of T-helper differentiation in vitro [14] and the resistance of mature murine T-cells to neoplastic transformation [15]. Additionally, GATA3 has been observed to function as a proto-oncogene in many T-cell tumor types, including precursor neoplasms, without concurrent expression of other Th2-specific genes, further challenging the ontological relationship between GATA3-expressing PTCLs and Th2 cells [16].

We observed several features in cases of canine CD4+ PTCL that suggest a more immature cell of origin than the conventionally speculated mature CD4+ T-helper cell phenotype. First, canine CD4+ PTCL cells exhibited low surface MHC class II expression by flow cytometry and significantly decreased transcriptomic expression of MHC class II-associated genes compared to control canine CD4+ lymphocytes. Unlike in humans, expression of MHC class II molecules is a feature of all peripheral T cells in dogs, although its expression in the neonatal thymus and on resting T cells is weaker than on activated T cells [57, 58]. Thymocytes collected from the thymuses of normal healthy control dogs in our study demonstrated significantly lower expression of MHC class II by flow cytometry compared to CD4+ T-cells from normal lymph nodes. This could suggest that the low MHC class II expression in canine CD4+ PTCL may be a feature of their relative immaturity.

Canine CD4+ PTCL cells also lacked surface expression of CD25 by flow cytometry, which was corroborated by significantly decreased expression of the *IL2RA* gene compared to control nodal CD4+ T-cells. CD25 is the alpha chain of the IL-2 receptor, which heterodimerizes with the beta and gamma chains to result in high-affinity binding of the receptor to IL-2 and promotion of lymphocyte proliferation and survival [59]. In humans and dogs, CD25 expression is associated with an activated lymphocyte phenotype under normal conditions [60–62], although it has also been expressed in a number of lymphoid neoplasms in both species [7, 63, 64]. In human thymocytes, CD25 is transiently expressed in early intrathymic progenitors and early subsets of double-negative (DN) thymocytes, although later subsets of DN thymocytes, DP thymocytes, and most SP thymocytes (with the exception of a portion of CD4 SP thymocytes that go on to differentiate into regulatory T-cells) lack surface expression of CD25 [65]. In our study, canine DP thymocytes lacked surface expression of CD25, and only a small proportion of canine DN thymocytes were CD25+ by flow cytometry. However, a significantly higher proportion of CD25+ cells were observed in the CD4 SP thymocytes and normal nodal CD4+ T-cell populations. Thus, the low surface CD25 expression observed in canine CD4+ PTCL in our study could indicate a tumorigenesis event somewhere around the time of DN or DP thymocyte development.

Although none of the PTCLs in this study expressed cell surface CD34 by flow cytometry, distinguishing them from T-cell acute lymphoblastic leukemia (T-ALL) [66], several genes associated with immaturity or early T-cell development were upregulated in the transcriptome. CD4+ PTCL samples compared to control canine CD4+ lymphocytes showed increased expression of *KIT*, *DNTT*, *CD34*, and *CCR9*. *KIT* is a proto-oncogene that encodes a transmembrane tyrosine kinase and plays an important role in hematopoietic stem cell (HSC) proliferation, differentiation, and survival, and is subsequently downregulated upon HSC maturation [67]. *DNTT* encodes terminal deoxynucleotidyl transferase (TdT), whose expression is normally restricted to immature lymphocytes in the thymus and bone marrow [68], but is also a frequent feature of acute lymphoid leukemias [69]. *CD34* is widely utilized as a marker of hematopoietic progenitor cells in dogs [70]. *CCR9* encodes a chemokine receptor whose expression is restricted to T cells in the thymus or small intestine, and in the thymus, it appears to play a role in T-cell development and migration [71, 72]. CCR9 is expressed by most DP thymocytes but not DN thymocytes, and it is subsequently downregulated with the transition from the DP stage to SP stages of thymocyte development [71]. Interestingly, while not described as a typical feature of the gene expression profile of human PTCL-NOS, we did observe similar upregulation of *CD34* in the transcriptome of human PTCL-NOS based on publicly available gene expression data from 68 cases of PTCL-NOS published by Etebari et al. [33] (log2 fold change = 5.15, padj = 1.53 × 10^-26^) and 28 cases of PTCL-NOS published by Piccaluga et al. [32] (log2 fold change = 0.46, padj = 5.08 × 10^-01^). Additionally, a gene expression profiling study by Helgeland et al. [31] found significantly increased expression of *CD34*, *DNTT*, and *CCR9* in CD4 SP thymocytes compared to circulating CD4+ T-cells in infant and adult peripheral blood, suggesting that the upregulation of these genes in canine CD4+ PTCL could favor a thymic precursor cell of origin over a more mature naïve or activated CD4+ T-cell phenotype.

GSEA utilizing our gene expression data revealed significant negative enrichment of canine CD4+ PTCL for gene signatures associated with TCR signaling, suggesting that TCR signaling may be downregulated (or not yet upregulated) in canine CD4+ PTCL. Taken together, the decreased expression of CD25 and MHC class II-associated genes, increased expression of various markers of immaturity and early T-cell development, and downregulation of TCR signaling suggest a naïve or precursor T-cell phenotype as a more likely cell of origin for canine CD4+ PTCL than an activated mature CD4+ T-cell, despite the upregulation of Th2-associated transcription factor GATA3.

The gene expression profile of canine CD4+ PTCL in our study was enriched for human gene signatures associated with early T-lymphocytes, intrathymic T progenitor cells, CD4 SP thymocytes, and DP thymocytes compared to naïve circulating CD4+ T-cells. There was no significant enrichment for human or murine Th17 or regulatory T-cell signatures in canine CD4+ PTCL compared to control CD4+ lymphocytes. GSEA using microarray data from one study (GSE14308) found no significant enrichment of our canine CD4+ PTCL gene expression data for murine Th1 or Th2 gene signatures, although a contradictory study (GSE22886) suggested enrichment for both activated human Th1- and Th2-associated gene signatures in canine CD4+ PTCL.

GSVA evaluating the enrichment of individual samples for the expression profiles of various stages of thymocyte development, resting and activated human immune cells, murine T-helper cell subsets, and the top 250 upregulated and downregulated genes in human CD4 SP infant thymocytes compared to CD4+ T-cells in adult and infant blood (from Helgeland et al. [31]) found that while control canine CD4+ lymphocytes were uniformly negatively enriched for early T-cell progenitor and thymocyte gene signatures and strongly enriched for naïve T-cell signatures, canine CD4+ PTCL samples appeared to split into two distinctive groups. The first group was characterized by strong enrichment of tumor cells for early T-lymphocyte gene signatures, intrathymic T progenitor cells, and genes upregulated in DP thymocytes compared to naïve circulating CD4+ T-cells and variable enrichment for CD4 SP thymocyte gene signatures, while also seeming to be enriched for genes upregulated with Th1 and Th2 cell activation despite this upregulation of early thymocyte gene signatures. Interestingly, all the Boxers in our study—a breed that is overrepresented for PTCL in dogs—clustered into this group, which could suggest a similar genetic profile for PTCL among members this breed. Tumors of the second group were largely negatively enriched or non-enriched for early T-lymphocyte and intrathymic T progenitor cell signatures, but still showed positive enrichment for CD4 SP thymocyte gene signatures compared to mature circulating CD4+ T-cells and enrichment for genes upregulated in naïve CD4+ T-cells compared to activated Th1 and Th2 cells. This distinctive clustering within our canine CD4+ PTCL cases may suggest that while the tumors in our study exhibited several features to suggest a more immature cell of origin, these neoplastic T-cells may still retain some ability to differentiate, leading to the formation of subtypes reflecting varying degrees of T-cell differentiation. Additional gene expression profiling studies of canine CD4+ PTCL are needed to support this conclusion.

Despite the increased expression of markers of immaturity in the transcriptome of canine CD4+ PTCL, the enrichment of these tumor cells for early thymocyte gene signatures, and downregulation of TCR signaling, canine CD4+ PTCL cells do still express mature T-cell surface antigens CD3 and CD5, although surface expression of the latter may be variable. They also exhibit clonal rearrangement of their TCR and downregulation of *RAG1* and *RAG2* genes (Supplementary Table 2B), features more typical of post-thymic T cells, but they downregulate the expression of genes associated with TCR signaling and are not enriched for gene signatures associated with naïve or mature peripheral T-cell phenotypes. The reason for these discrepancies is unclear. T-cell malignancies that originate from thymic precursors but still express a TCR have been reported in the human literature. For example, in anaplastic large cell lymphoma (ALCL), TCR rearrangement analyses identified malignant transformation of tumor cells in thymocytes prior to TCR β-rearrangement, but RAG-independent TCR expression driven by the oncogenic NPM-ALK fusion protein still allowed these cells to exit the thymus, and downregulation of the TCR was required for subsequent lymphomagenesis [73]. The negative enrichment of canine CD4+ PTCL for the TCR signaling pathway and upregulation of various markers of immaturity could suggest a similar process in canine CD4+ PTCL, although similar oncogenic gene fusions have yet to be investigated in this disease. Another possibility could include the reversion of mature CD4+ T-cells to a more immature phenotype following malignant transformation. Primary human CD4 T cells transduced with the previously mentioned *NPM-ALK* fusion have been shown to adopt a more immature gene expression profile and undergo malignant transformation following TCR stimulation with anti-CD3/28 [74]. In mice, *PAX5* deletions have been shown to result in reversion of mature B cells to an uncommitted precursor cell phenotype and induce the formation of progenitor cell lymphomas [75]. A similar driver mutation in canine PTCL could be driving the dedifferentiation of mature CD4+ T-cells to a more immature thymic precursor phenotype. Additional studies investigating driver mutations in canine CD4+ PTCL may help identify mechanisms contributing to the contradictory immature tumor cell transcriptome with surface expression of mature T-cell markers.

A major limitation of our study was the reliance on human and murine gene sets for evaluating enrichment of canine CD4+ PTCL for thymocyte, naïve T-cell, mature T-cell, and oncogenic pathway gene signatures due to the relative paucity of published gene expression data for their canine counterparts. Future studies utilizing gene expression data from the canine counterparts of these T-cell lineages may help more confidently elucidate the cell of origin of canine CD4+ PTCL. Additionally, while we have demonstrated the dependence of canine CD4+ PTCL on PI3K for survival and proliferation, future studies assessing the dependence of these tumors on more downstream effectors within this pathway are warranted to refine the role of PI3K/AKT signaling pathway in canine CD4+ PTCL.

## Conclusions

Our findings indicate that canine CD4+ PTCL is a molecularly comparable disease to human PTCL-NOS with a similar gene expression profile. Given the increased expression of GATA3, downregulation of PTEN, enrichment for PI3K/AKT/mTOR signaling, and in vitro dependence on this pathway for tumor cell survival and proliferation, canine CD4+ PTCL appears to most closely resemble the GATA3-PTCL subtype of human PTCL-NOS, which may allow for a more specific application of the proposed canine model for this disease.

Additionally, our study suggests that canine CD4+ PTCL may arise from a thymic precursor cell of origin, based on the observed enrichment for gene signatures associated with early thymocyte progenitors, downregulation of TCR signaling, decreased expression of CD25 (IL2RA) and MHC class II-associated genes, and increased expression of markers of immaturity. This has important implications for the pathogenesis of canine PTCL, as this precursor-like gene expression profile with downregulation of TCR signaling could suggest that chronic inflammation leading to prolonged TCR stimulation is likely not implicated as a predisposing factor for the development of canine PTCL.

### Supplementary Information

**Additional file 1:** **Supplementary Fig. 1.** A Tukey’s multiple comparisons test revealed significantly lower surface MHC class II expression (measured as median fluorescence intensity) in all canine PTCL phenotypes compared to control nodal CD4+ T-cells (p>0.0001 for CD4+ PTCL and DN PTCL, and *p*=0.0316 for CD8+ PTCL).

**Additional file 2:** **Supplementary Fig. 2.** Principal component analysis (PCA) of all samples in our study revealed an outlier (CF21) whose variation in the second principal component dimension that was >3x the standard deviation of all samples in the CD4+ PTCL group. This outlier was subsequently removed from further gene expression and gene set enrichment analyses.

**Additional file 3:** **Supplementary Table 1A. **Signalment and clinical features of dogs in this study. **Supplementary Table 1B. **Flow cytometric features of canine PTCLs. **Supplementary Table 1C. **Flow cytometric features of normal control nodal and circulating lymphocytes. **Supplementary Table 1D. **Flow cytometric features of normal control canine thymocytes. **Supplementary Table 2A. **Top 500 differentially expressed genes in canine CD4+ PTCL compared to control canine CD4+ lymphocytes. **Supplementary Table 2B. **Normalized RNA-seq read counts. **Supplementary Table 3A. **GSEA results for MSigDB gene sets using the Broad Institute GSEAPreranked tool. **Supplementary Table 3B. **GSEA results for curated gene sets using the Broad Institute GSEAPreranked tool. **Supplementary Table 4A. **GSEA results for MSigDB gene sets using clusterProfiler. **Supplementary Table 4B. **GSEA results for curated gene sets using clusterProfiler. **Supplementary Table 5. **GSVA scores. **Supplementary Table 6A. **Novogene RNA-seq QA/QC report. **Supplementary Table 6B. **MultiQC RNA-seq QA/QC report. **Supplementary Table 7. **Software programs and packages. **Supplementary Table 8. **Correlation of protein expression by flow cytometry and gene expression by RNA-seq.

## Data Availability

The datasets used and analyzed during the current study are available from the corresponding author on reasonable request.

## Abbreviations

ALCL: Anaplastic large cell lymphoma
DMSO: Dimethyl sulfoxide
DP: Double-positive
GSEA: Gene set enrichment analysis
IHC: Immunohistochemistry
MFI: Median fluorescence intensity
NES: Normalized enrichment score
PCA: Principal component analysis
PI: Propidium iodide
PTCL: Peripheral T-cell lymphoma
PTCL-NOS: Peripheral T-cell lymphoma, not otherwise specified
RNA-seq: RNA sequencing
SP: Single-positive
T-ALL: T-cell acute lymphoblastic leukemia
TCR: T-cell receptor
