# Supplementary figures and images for: The gene expression profile and cell of origin of canine peripheral T-cell lymphoma
Source: BMC Cancer. 2024 Jan 2;24:18. doi: 10.1186/s12885-023-11762-w (PMC10762913; doi:10.1186/s12885-023-11762-w)

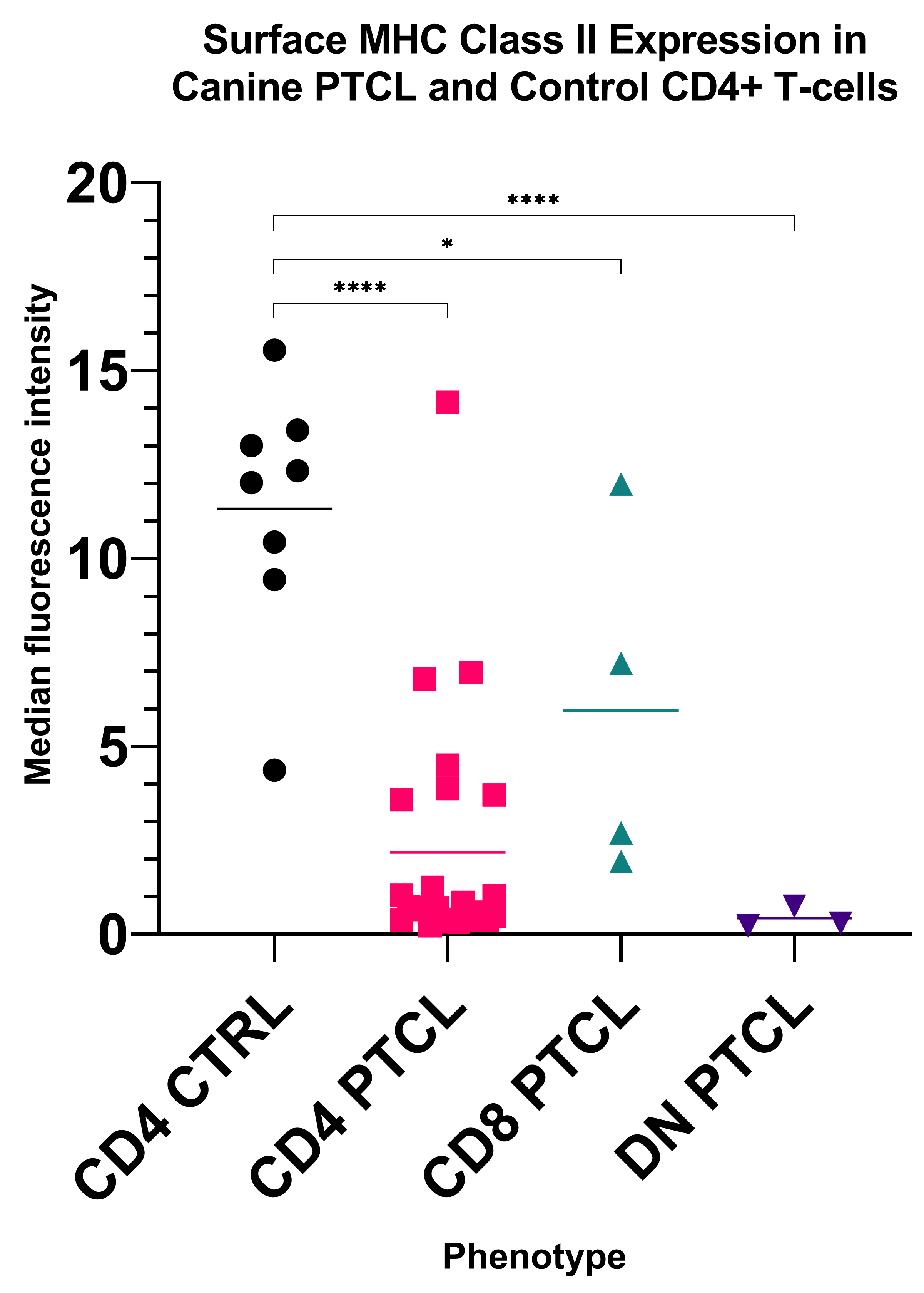

Supplement: Supplementary file 1 — Additional file 1: Supplementary Fig. 1. A Tukey’s multiple comparisons test revealed significantly lower surface MHC class II expression (measured as median fluorescence intensity) in all canine PTCL phenotypes compared to control nodal CD4+ T-cells (p>0.0001 for CD4+ PTCL and DN PTCL, and p=0.0316 for CD8+ PTCL). [file 12885_2023_11762_MOESM1_ESM.png]

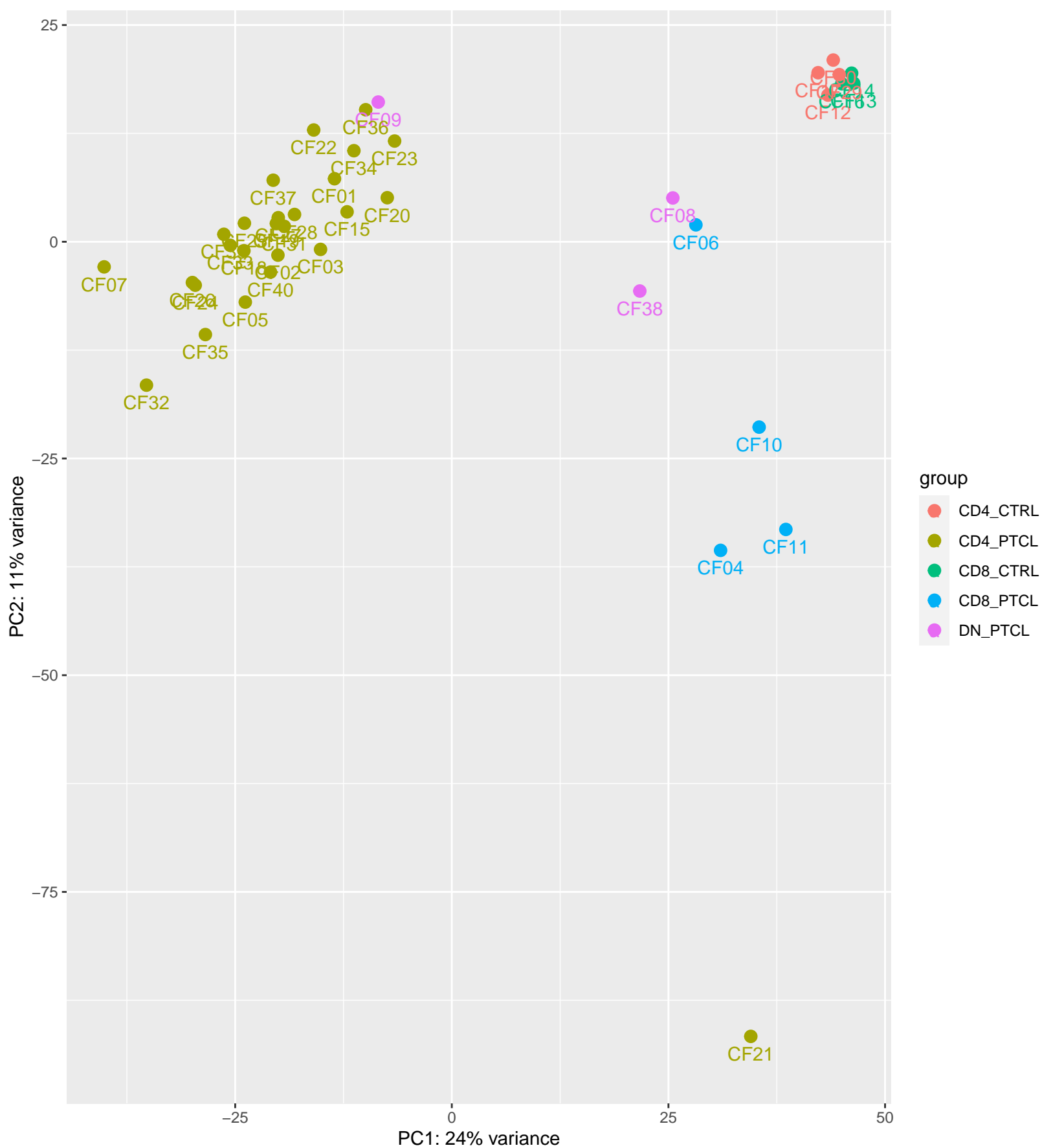

Supplement: Supplementary file 2 — Additional file 2: Supplementary Fig. 2. Principal component analysis (PCA) of all samples in our study revealed an outlier (CF21) whose variation in the second principal component dimension that was >3x the standard deviation of all samples in the CD4+ PTCL group. This outlier was subsequently removed from further gene expression and gene set enrichment analyses. [file 12885_2023_11762_MOESM2_ESM.pdf]
